# Invasive Orbital Squamous Cell Carcinoma in a Patient with Multiple Myeloma

**DOI:** 10.1155/2022/8585692

**Published:** 2022-06-29

**Authors:** Narges Karrabi, Kiana Hassanpour, Noushin Afshar Moghaddam, Faezeh Khorasanizadeh, Sadid Hooshmandi, Amirreza Veisi

**Affiliations:** ^1^Ophthalmic Research Center, Research Institute for Ophthalmology and Vision Science, Shahid Beheshti University of Medical Sciences, Tehran, Iran; ^2^Department of Ophthalmology, Imam Hossein Hospital, Shahid Beheshti University of Medical Sciences, Tehran, Iran; ^3^Department of Pathology, Imam Hossein Hospital, Shahid Beheshti University of Medical Sciences, Tehran, Iran; ^4^Department of Radiology, Razi Hospital, Tehran University of Medical University, Tehran, Iran

## Abstract

**Background:**

Orbital squamous cell carcinoma (SCC) is a rare entity. It is often a result of local invasion of SCC originating from the skin, nasopharynx, nasal cavity, paranasal sinuses, conjunctiva, lacrimal glands, or sac or less commonly occurs through hematogenous metastasis. Herein, we report a patient with orbital SCC with a history of multiple myeloma (MM). *Case presentation*. A 45-year-old woman with a history of MM in the past two years presented to our clinic complaining of gradual right eye proptosis for six months. The relative afferent pupillary defect was detected in the right eye on her examination. Ocular movements of the right eye were limited in all directions. Orbital magnetic resonance imaging demonstrated an infiltrative mass in the right orbit extended from the anterior to the orbital apex and the optic canal. The patient underwent debulking, and a histopathology examination revealed SCC results. No other secondary site was found to be the origin of the tumor.

**Result:**

The patient underwent chemotherapy and subsequent radiotherapy. To our knowledge, this is the first report of concomitant MM and primary orbital SCC.

## 1. Introduction

Orbital squamous cell carcinoma (SCC) is an infrequent entity [1–6]. The most probable origins of orbital SCC include local invasion along with perineural spread (PNS) from lesions of adjacent periorbital skin, paranasal sinuses, and ocular adnexa. However, it can arise from the choristomatous squamous epithelium and metastasis from a far site through hematogenous spread [7]. The classical features resemble to other orbital lesions and include orbital proptosis, diplopia, limitation of the eye movement, and compressive neuropathy [4–6].

Multiple myeloma (MM) is a neoplastic proliferation of plasma cells and the second most common blood cancer (10%) after non-Hodgkin lymphoma (NHL). Various body organs may be affected in patients with MM including the bone, skin, and kidney. Patients' signs and symptoms vary greatly based on different involved organs. However, the bone pain, impaired kidney function, and anemia occur frequently [8]. In a patient with MM, orbital involvement has also been described and may occur primarily due to invading neoplastic cells from bony orbital walls to orbital soft tissues or second primary malignancies (SPMs) such as lymphoma. Moreover, metastasis from distant SPMs can be another source of orbital tumors [9].

To our knowledge, less than 15 cases with orbital SCC have been currently reported [1–3]. Herein, we report orbital SCC in a patient with MM. To our knowledge, this is the first report of orbital SCC in a patient with MM.

## 2. Case Presentation

**Figure 1:**
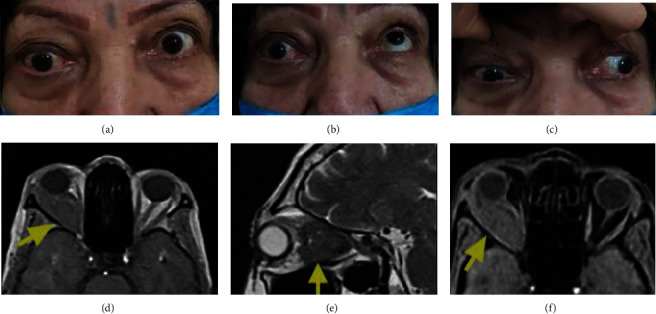
A 45-year-old female presented with proptosis, periorbital fullness, and movement limitation in the right eye (a–c). Orbital MRI demonstrates a large orbital mass in the lateral part of the right orbit that spreads to the orbital apex and pushes the optic nerve medially. The mass is isointense to the brain tissue in T1- (d) and T2-weighted (e) images and enhances homogeneously in T1-weighted fat saturation (f).

A 45-year-old woman with a history of MM treated by bortezomib and zoledronic acid in the past two years presented gradually exacerbating blurred vision, outward protrusion of the right eye, and double vision since six months prior. The patient also noted dull right orbital pain and constant headache. At presentation, the best-corrected visual acuity (BCVA) was 20/400 and 20/40 (Snellen chart) in the right and the left eye, respectively. A grade 3 afferent pupillary defect was detected in the right eye. She had decreased sensation of V1 dermatome on the right side of her face. On examination, the right eye had nonaxial proptosis assessed by Hertel exophthalmometer and limitation in the movements, particularly lateral and upward rotation (Figure 1). The eyelid examination revealed no pathologic findings. On the slit-lamp examination, the cornea and anterior segment were normal. A moderate nuclear cataract was observed in both eyes. Intraocular pressure was16 mmHg in the right eye and 13 mmHg in the left eye. The optic nerve head in the right eye had a diffuse pallor. Other fundus examinations were normal. The color test using pseudoisochromatic Ishihara plates revealed abnormal results in the right eye (3/14 plates) and normal results in the left eye.

Magnetic Resonance Imaging (MRI) revealed an infiltrative extra and intraconal mass primarily located in the lateral part of the orbit. The mass spreads to the medial portion and the apex of the right orbit. The orbital mass was isointense and mildly hypointense in the T1-weighted and T2-weighted images. Postcontrast T1-weighted images disclosed remarkable enhancement of the mass (Figure 1). No paranasal sinus involvement was evident.

**Figure 2:**
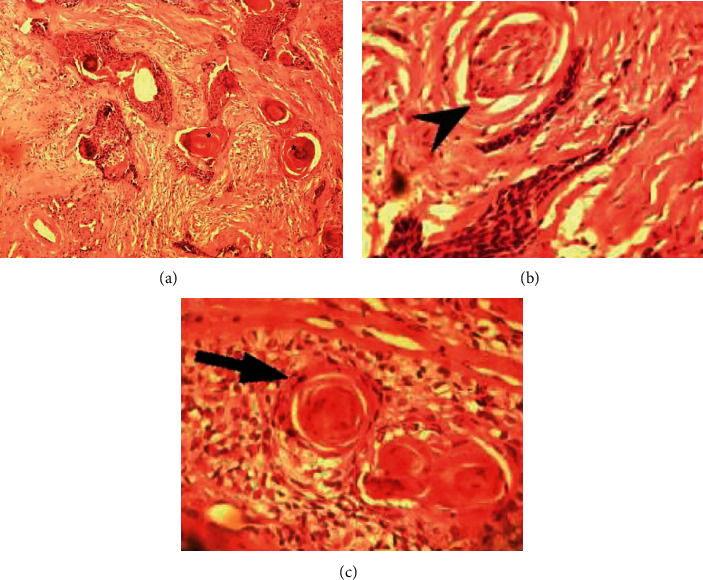
(a) Photomicrograph of orbit with well-differentiated squamous cell carcinoma showing an infiltrative growth pattern of epithelial cells with some keratin pearls (asterisk) (low power field, ×100, H&E staining). (b) Photomicrograph from orbital well-differentiated squamous cell carcinoma revealing squamous neoplastic epithelial cells with intercellular desmosomes and keratin pearls (arrowhead) (high power field, ×400, H&E staining). (c) Photomicrograph from the perineural invasion of orbital squamous cell carcinoma (arrow) (medium power field, ×200, H&E staining).

The patient underwent lateral orbitotomy. Due to tumor extension to the optic canal, total resection was impossible, and only a conservative tumor debulking was performed. Histopathology exam exhibited a neoplastic proliferation of squamous cells arranged in nests and sheets embedded in the desmoplastic stroma. These cells had a high nucleus/cell ratio with moderate atypia and pleomorphism. Tumor necrosis was also observed. Lacrimal gland admixed with fibrotic tissue of tumor was noted. The histopathologic findings were consistent with a well-differentiated invasive SCC, mixed small and large cell keratinizing type (Figure 2).

Systemic workup consisting of dermatologic and otolaryngologic consult, nasal/sinus endoscopy, and full-body computed tomography revealed no extra orbital malignancy. According to the orbital apex and optic canal involvement, the patient received 3 cycles of chemotherapy with 5-fluorouracil (800 mg/m2/d i.v. on days 1-5) and cisplatin (50 mg/m2/d i.v. on days 1-5) and radiation therapy (intensity-modulated radiation therapy 5040 cGY) in the first step. The patient was planned for palliative exenteration, and she denied the treatment. Two months after the orbitotomy and the start of chemotherapy and radiation, the proptosis has been partially resolved, although ocular movements and the BCVA were the same as presentation.

The study adheres to the tenets of the Declaration of Helsinki. The patient's permission was obtained to publish her data.

## 3. Discussion

Herein, we reported an orbital SCC in a patient with MM. The orbital tumor can be considered primary; however, the definite differentiation between a primary essence and a local invasion from the lacrimal gland is impossible because of the large tumor extension. Since the orbit typically lacks squamous epithelium, primary orbital SCC is infrequent. To our knowledge, less than 15 cases have been currently reported [1–3, 7]. It mainly occurs due to local invasion of SCC of the skin or nasopharynx, nasal cavity, paranasal sinuses, conjunctiva [7], lacrimal glands [10], or sac [10]; occult extension by perineural spread (PNS) [11]; or hematogenous metastasis. Metastatic tumors are usually poorly differentiated, with little keratin, and they generally have a poor prognosis, with the patient expiring within a few months or a year [10]; in our case, histopathologic examination demonstrated a large amount of keratin against the metastatic origin of the disease, and systemic investigations did not reveal any source responsible for metastasis.

Furthermore, orbital SCC may arise from orbital choristomatous squamous epithelium [1]. Few authors have reported orbital SCCs arising from epithelial inclusion cyst, epidermoid [12] and dermoid cysts [13], and lacrimal gland cysts with squamous metaplasia [14].

A possible route is iatrogenic transplanted conjunctival epithelium following a scleral buckling surgery and consequent malignant transformation long-term after the procedure [15]. A relatively common way of orbital SCC is the PNS of the tumor described by Cruveilhier [16]; various tumors such as adenoid cystic carcinoma of the lacrimal gland [17], cutaneous tumors including SCC and BCC [18], and rarely melanoma may behave in this manner. Mohs and Lathrop first described the involvement of the orbit by PNS of a cutaneous SCC in 1909 [19]. Reed and Leonard [20] encountered two categories of patients with PNS. First, the patients involved in the ocular adnexa or orbit by distant PNS extending beyond the index lesion, usually located elsewhere on the face or scalp. Second, microscopic PNS was incidentally found on histologic examination of a lesion excised from the ocular adnexa [21]. McNab et al. [22] have reported the PNS of cutaneous SCC into orbit. They said 21 cases with small and distinct primary cutaneous lesions. PNS development interval ranges from 5 weeks to 10 years. Patients may have a destroyed skin lesion that they could not remember, resulting in occult PNS. Cranial nerve (CN) VII involvement is a common finding with PNS. Radiologic features suggestive of PNS are nerve or foramina enlargement/enhancement, obliteration of fat planes, and pseudocystic masses. MRI and CT have a sensitivity of 76% to 95% [22]. Hence, negative imaging results do not exclude PNS. Our patient lacked any source of PNS of cutaneous tumors both by examination and history. Facial nerve function was normal and radiologic findings were not in line with PNS.

Regarding reported patients in the literature, the interval between the onset of symptoms and diagnosis of SCC may range from less than two months [2] to 18 years [3]. No sex predilection has been reported. The average age of patients was 59.7 years (range 43-74). The most common complaints leading to medical evaluations included blurry vision, ophthalmoplegia and diplopia, eyelid ptosis with or without edema, pain (ocular, facial, periocular, or headache), discharge, red eye, V1 or V2 paresthesia, and even total lack of ocular signs.

Various evaluations, including orbital and brain CT or MRI, incisional and excisional biopsy, full-body CT, or PET scan, are generally used to assess the diagnosis and investigate the primary source for SCC. The most common reported site is the orbital apex, followed by the superomedial and superolateral parts of the orbit. The involvement of inferior intraconal space between the inferior rectus and optic nerve has also been reported.

Our patient is unique because of the prior diagnosis of MM. Different population-based studies have reported increased incidence of SPM in patients with MM, particularly for acute myeloid leukemia and NHL, and a slight increase of both melanoma and nonmelanoma skin cancers. Interestingly, decreased risk of solid tumors was noted by several studies [23]. SPMs mostly appear to start 12 months after MM diagnosis, and the risk increases with time, with the highest rates usually seen after 5–10 years [24]. Being multifactorial, the combination of intrinsic and extrinsic risk factors plays a role in SPM. Intrinsic risk factors include biologic-related factors such as male gender, advanced age, African American race, comorbidities, and genetic predispositions and disease-related factors such as complex/high-risk cytogenetics vs. favorable cytogenetics in patients with long-term disease [25].

Extrinsic risk factors include treatment regimen, disease duration, environmental, and lifestyle factors like smoking, sun exposure, and obesity [25]. Regarding treatment regimen and its duration, melphalan [26] and lenalidomide increase the risk of hematologic SPM [27]. Indeed, autologous stem cell transplantation (ASCT) may increase the risk of hematologic malignancies and some solid tumors, specifically the skin and colon cancer [28]. Our case was treated with neither the drugs above nor ASCT. Bortezomib treated her for two years, and several studies have noted its safety and lack of risk of SPM development [29].

Additionally, MM can involve the orbit with slow infiltration and an insidious progression, mostly (65%) in patients with a prior diagnosis of MM averagely within 17.6 last months. The mass is typically situated in the extraconal posterior orbit, especially in the superotemporal quadrant [30]. In our patient, despite an initial clinical diagnosis of orbital MM or plasmacytoma, further investigations disclosed an orbital SCC with a presumably primary origin. To the best of our knowledge, no similar report exists based on a widespread literature review. There is only a case report of primary orbital SCC and the previous history of lymphoma. However, the author hypothesized malignant transformation of implanted conjunctival epithelium surrounding the site of the last scleral explant to be responsible for orbital SCC [31]. It is not apparent that MM and chemotherapy regimens predispose the patient for an orbital SCC, or this association is just an extremely rare coincidence. Further similar reports will enhance our knowledge about unknown aspects of this rare orbital tumor.

Our patient underwent an excisional biopsy followed by chemoradiotherapy. The standard treatment regimen is still unclear due to scant published data addressing this uncommon situation; however, it seems the mainstay of treatment remains orbital radiation. Different treatment strategies have been proposed, including orbital radiation, orbital exenteration, adjuvant radiotherapy, orbital radiation, and adjuvant chemotherapy [1–3].

Similarly, the prognosis of patients with orbital SCC is indeterminate. Peckinpaugh et al. reported fellow orbit involvement in one patient and enucleation of the eye of the involved orbit due to neovascular glaucoma [1]. Both patients expired after 12 and 19 months [1]. One report of recurrence after initial surgery and adjuvant radiotherapy exists, which led to palliative exenteration, and the patient eventually expired two years after the onset of symptoms [12]. Other reports noticed variable surveillance following treatment at least for a period ranging from 12 months to 7 years at the time of reporting their cases [2, 15, 31], but the ultimate prognosis of patients is not clarified. Accordingly, primary orbital SCC appears to be highly mortal despite treatment; early aggressive treatment may be warranted.

## Data Availability

The data are available in the article or on request. Please contact Dr. Kiana Hassanpour (kiana.hassanpour@gmail.com).
